# Acute High Dose Melatonin for Encephalopathy of the Newborn (ACUMEN) Study: a protocol for a multicentre phase 1 safety trial of melatonin to augment therapeutic hypothermia for moderate/severe hypoxic ischaemic encephalopathy

**DOI:** 10.1136/bmjopen-2025-107083

**Published:** 2025-08-22

**Authors:** Raymand Pang, Alyson Macneil, Anvi Wadke, Yusuf Jaami, Neil Marlow, Joseph F Standing, Hakim-Moulay Dehbi, Pamela Tranter, Nicola J Robertson, on behalf of the ACUMEN Consortium, Karel Allegaert

**Affiliations:** 1 EGA Institute for Women’s Health, University College London, London, UK; 2 Comprehensive Clinical Trials Unit, University College London, London, UK; 3 Joint Research Office, University College London Hospitals/University College London, London, UK; 4 Great Ormond Street Institute of Child Health, University College London, London, UK; 5 Translational Research Office, University College London, London, UK

**Keywords:** Hypoxia, Neonatology, Clinical Trial, Brain Injuries

## Abstract

**Introduction:**

Neonatal death and later disability remain common sequelae of hypoxic-ischaemic encephalopathy (HIE) despite the now standard use of therapeutic hypothermia (HT). New therapeutic approaches to brain protection are required. Melatonin is an indolamine hormone with free-radical scavenging, antiapoptotic, anti-inflammatory and gene regulatory neuroprotective properties, which has extensive preclinical evidence of safety and efficacy. Pharmacokinetic (PK) data suggest it is necessary to reach melatonin levels of 15–30 mg/L within 6–8 hours of hypoxia-ischaemia for brain protection. We developed a novel Good Manufacturing Practice (GMP) grade melatonin in ethanol 50 mg/mL solution for intravenous use. In preclinical studies, ethanol is an adjuvant excipient with additional neuroprotective benefit; optimised dosing protocols can achieve therapeutic melatonin levels while limiting blood alcohol concentrations (BACs).

**Methods and analysis:**

The Acute High Dose Melatonin for Encephalopathy of the Newborn (ACUMEN) Study is a first-in-human, international, multicentre, phase 1 safety study of intravenous melatonin in babies with moderate/severe HIE receiving HT. Sixty babies will be studied over two phases: a dose escalation study including four dose levels to establish the recommended phase 2 dose (RP2D), followed by a 6-month cohort expansion study of RP2D to further characterise PKs and affirm safety. Participants will receive a 2-hour intravenous infusion of melatonin within 6 hours of birth, followed by five maintenance doses every 12 hours to cover the period of HT. Plasma melatonin and BACs will be monitored. The RP2D will be based on the attainment of therapeutic melatonin levels while limiting BACs and the frequency of dose-limiting events (DLEs). A Bayesian Escalation with Overdose Control approach will be used to estimate the risk of DLE per dose level, with a target level of <33%. ACUMEN will establish a network of centres with standardised neurocritical care and harmonised MRI systems for the analysis of the primary outcome—magnetic resonance spectroscopy (MRS) lactate to N-acetylaspartate peak area ratio localised to the basal ganglia and thalamus and include a nested blood biomarker study to explore early disease severity indicators.

**Ethics and dissemination:**

Approval has been given by the London Central National Health Service Health Research Authority Ethics Committee (25/LO/0170) and UK Clinical Trials Authorisation from the Medicines and Healthcare products Regulatory Agency. Separate approvals have been sought in Ireland and Australia. Dissemination will be via peer-reviewed journals, conference presentations, public registries and plain language summaries for parent/legal guardian(s), in accordance with national requirements.

**Trial registration number:**

ISRCTN61218504. EU CT: 2025-520538-49-00.

**Protocol version:**

Publication based on the UK protocol V.3.0, 08 May 2025

**STRENGTHS AND LIMITATIONS OF THIS STUDY**

- The Acute High Dose Melatonin for Encephalopathy of the Newborn (ACUMEN) Trial is a phase 1 safety and dose escalation study which will establish the recommended phase 2 dose of a novel melatonin formulation in the target high-risk population of babies with moderate/severe hypoxic-ischaemic encephalopathy (HIE) who are being treated with therapeutic hypothermia, a treatment which is only partially effective.
- Dose escalation decisions will be contingent on safety and established target melatonin levels, based on robust safety, efficacy and pharmacokinetic preclinical data.
- The ACUMEN Trial uses a Bayesian Escalation with Overdose Control approach to estimate risk of dose-limiting events with accumulating trial safety data to support dose escalation decisions.
- A network of 10 large, tertiary neonatal units across the UK, Ireland and Australia, with experience of recruiting into neonatal and HIE trials and a standardised level of neurocritical care, will establish a network for future phase 2 trials.
- The requirement for the loading dose to be administered within 6 hours is necessary but challenging in this multicentre study.

## Introduction

### Background and clinical need

 Hypoxic-ischaemic encephalopathy (HIE) is a leading cause of neonatal mortality and morbidity, affecting 2–3 babies per 1000 live births in high-income countries.^1^ Therapeutic hypothermia (HT) is currently the only treatment available for moderate/severe HIE, and, while partially effective, babies still develop an unacceptable level of long-term neurological sequelae. Almost two decades since the initial cooling trials and adoption as standard care for moderate/severe HIE in 2010 in the UK, there has been limited progress to further improve outcomes. Recent data highlight that, despite HT, more than 50% of babies die or develop neurodevelopmental impairment, including 10%–19% with cerebral palsy.^2 3^ Long-term neurological sequelae remain of concern despite treatment optimisation;^3^ up to 60% of children develop neurocognitive, behavioural or executive processing problems, impacting learning.^4^ Importantly, follow-up of the original cooling trial cohorts shows that persistent neurological impairment later in childhood, impacting IQ, non-verbal performance score, processing speed, working memory and behaviour, is common.^5 6^

Further attempts to optimise cooling protocols with longer and deeper cooling did not improve outcomes^3^ and new therapies are urgently needed. Promising therapies such as inhaled Xenon^7^ and Erythropoietin^2^ have not demonstrated treatment benefit in phase 2 and 3 trials, respectively, despite indications of potential benefit in large animal preclinical studies.^8^,^10^ A phase 3 randomised controlled trial to assess intravenous allopurinol with HT has been stopped early after recruiting 503 babies due to early indicators of lack of benefit of the intervention (EudraCT 2016-000222-19).

The Acute High Dose Melatonin for Encephalopathy of the Newborn (ACUMEN) Study is a first-in-human, international, multicentre phase 1 safety trial of melatonin to augment HT brain protection for moderate/severe HIE.

### Melatonin

Melatonin (N-acetyl-5-methoxytryptamine) is a promising therapy to translate from bench to cot side, with a pleiotropic profile for neuroprotection supported by comprehensive preclinical safety and efficacy data. The neuroprotective actions of melatonin include potent free radical scavenging properties, anti-inflammatory and antiapoptotic activities and gene regulatory effects on melatonin receptors (MT1 and MT2).^11^ These benefits inhibit the initiation of the injurious neurotoxic cascade leading to cell death during the period of secondary energy failure (from ∼6 hours after birth), thereby reducing adverse neurological outcomes.

While enteral forms of melatonin are readily available, the development of an intravenous formulation remains a key challenge. Melatonin has limited solubility in aqueous solution and the need to develop highly concentrated formulations acceptable for intravenous use in neonates necessitates the use of excipients, of which several have been used, in preclinical studies (including cyclodextrin, dimethyl sulfoxide and polysorbate). However, ethanol has shown the most favourable safety profile given its adjuvant, partial neuroprotective effects observed in animal models of HIE (in addition to HT)^12 13^ and adult stroke.^14^,^17^

The investigational medicinal product (IMP) is a novel melatonin in ethanol (50 mg/mL) manufactured in accordance with Good Manufacturing Practice (GMP) under a manufacturing authorisation licence for intravenous administration. The IMP will require dilution in 5% glucose to a final melatonin concentration of 2.5 mg/mL for intravenous infusion. The dosing regimen for this study has been optimised to maintain therapeutic melatonin levels while limiting the accumulation of ethanol and its by-product, acetaldehyde, as discussed below.

### Preclinical data

A preclinical systematic review and meta-analysis of 14 animal studies of hypoxia-ischaemia (HI) at term-equivalent age^18^ was an important milestone towards clinical translation to babies with HIE, along similar lines as the evolving guidelines for clinical translation in adult stroke included in the Stroke Therapy Academic Industry Roundtable (STAIR) criteria.^19^

In summary, we observed: (1) A significant reduction in brain infarct size (pooled standardised mean difference (SMD) −2.05, 95% CI −2.93 to −1.16, p<0.001, n=110 animals assessed), (2) Improved neurobehavioural outcomes (SMD −0.86, 95% CI −1.23 to −0.53, p<0.001), (3) Reduction in cell death (SMD −0.60, 95% CI −1.06 to −0.14, p=0.006). The overall observed effect size in all three outcomes combined remained significant (SMD −0.92, 95% CI −1.26 to −0.58, p<0.001). Importantly, subgroup analysis demonstrated benefit of melatonin as an adjunct to cooling with SMD −0.89, 95% CI −1.63 to −0.16, p<0.001, compared with HT alone, giving confidence in the added benefit of melatonin to HT. The meta-analysis also demonstrated that studies using formulations containing ethanol were most effective (SMD −1.14, 95% CI −1.64 to −0.65, p<0.001). This is supported by the intermediate improvement in neurological outcomes in newborn piglets administered ~300 mg/kg ethanol following HI.^12^

Intravenous melatonin has demonstrated consistent evidence of brain protection in newborn piglets^812 20^,^22^ and fetal lamb^23 24^ models of HIE. The key studies in newborn piglets are summarised in online supplemental table S1. These large animal studies are translationally relevant; the newborn piglet has a similar gyrencephalic brain structure and brain development comparable to newborn babies.^25^ In addition, neurocritical care monitoring (continuous video amplitude-integrated electroencephalography (aEEG/EEG)) and magnetic resonance spectroscopy (MRS)) are used as robust biomarkers in the piglet studies, giving translational relevance. In term neonates with HIE, evolution of the aEEG background pattern and time to recovery conveys prognostic information.^26^ Recovery of aEEG/EEG to either continuous or discontinuous normal voltage is associated with better prognosis, with the maximum predictive time at 48–72 hours.^27^ The clinical importance and relevance of lactate to N-acetyl aspartate peak area ratio (Lac/NAA) is based on clinical studies of babies with HIE in whom basal ganglia and thalamus (BGT) proton (^1^H) MRS accurately predicts 2-year motor, cognitive and language neurodevelopmental outcomes.^28 29^

**Figure 1:**
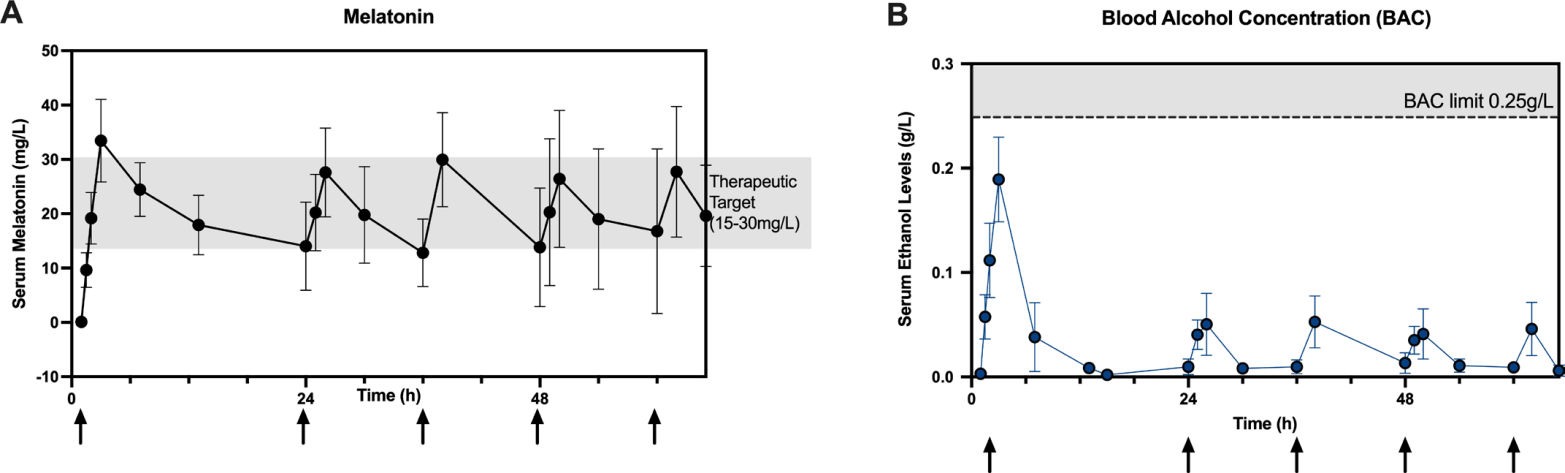
Melatonin and ethanol levels in 13 newborn piglets. Piglets received a loading of 20 mg/kg of melatonin over 2 hours (arrow) followed by four maintenance doses of 10 mg/kg over 2 hours every 12 hours from 24 hours. Target melatonin level (15–30 mg/L) (**A**) and BAC <0.25 g/L shaded (**B**).

Over the last 15 years the University College London (UCL) Neonatal Neuroprotection Research Group has focused on preclinical studies assessing safety, pharmacokinetics (PKs) and neuroprotective efficacy of intravenous melatonin in the newborn piglet; together, these studies give the foundation for the optimised dosing regimen in the clinical phase 1 ACUMEN Trial.^812 20^,^22^ These data suggest that the attainment of therapeutic melatonin levels of 15–30 mg/L within 6–8 hours of HI is necessary for optimal brain protection. Critically, a loading dose of 20 mg/kg is required to rapidly achieve therapeutic levels after birth, while 12-hourly maintenance doses of 10 mg/kg every 12 hours from 24 hours ensure therapeutic levels are maintained over time (see figure 1). This dosing regimen minimised blood ethanol (alcohol) concentrations (BACs), particularly during maintenance dosing (see figure 1B); we observed no evidence of accumulation of the alcohol by-product, acetaldehyde.^22^ These preclinical studies support the safety, PKs and efficacy of intravenous melatonin for clinical use in neonates.

No significant physiological safety concerns were observed with intravenous melatonin dissolved in ethanol. In study 1 (Table S1), there was no difference in mean arterial blood pressure during the 6-hour melatonin infusion and no difference overall in inotrope use.^20^ Most recently in study 5, there was no difference in mean arterial blood pressure, although we observed a clinically non-significant, mild increase in inotropic support following the loading dose (increase in vaso-inotropic score equivalent to a dopamine dose of 5 mcg/kg/hour).^22^ We observed no toxic effects of intravenous melatonin on haematology (full blood counts) and blood biochemical monitoring (liver function and renal function).

### Newborn lamb studies

The Monash Group demonstrated robust neuroprotection using a similar melatonin formulation containing ethanol at similar doses of 15–17 mg/kg^23 24^ but with a dosing regimen similar to a continuous infusion. Following umbilical cord occlusion, newborn lambs were administered melatonin 60 mg, dissolved in ethanol, given as 5 mg bolus intravenous injections every 2 hours from 30 mins after birth until 24.5 hours of life. Melatonin-treated animals showed a significant reduction in Lac/NAA, improvement in achieving neurobehavioural milestones and reduction in immunohistochemical markers of cerebral brain injury. There was no report of hypotension or need for increased inotropic support, although increased drowsiness was observed. Treatment benefit was additive to cooling, supporting the data from the newborn piglet studies.^24^ While this dosing regimen also shows promise, a 2-hour infusion used in the piglet studies may be more suitable in critically unwell infants.

### Clinical data

This is a first-in-human phase 1 clinical trial using a melatonin in ethanol formulation for intravenous use in HIE. To date, one other pilot study of intravenous melatonin administration has been conducted in this target population of term babies using a non-ethanol containing melatonin formulation.^30^ There are also limited pilot data of enteral administration of melatonin in this target population. These studies were underpowered with limited PK data reported, using high enteral doses up to 80 mg (~27 mg/kg).^31^

Jerez-Calero *et al* conducted the only clinical study of intravenous melatonin administration in babies with HIE receiving cooling.^30^ The melatonin formulation contained melatonin 6.5 mg/mL dissolved in propylene glycol and macrogol. Twelve babies with moderate/severe HIE undergoing HT received a daily dose of 5 mg/kg melatonin administered within 6 hours of birth intravenously over 2 hours every 24 hours for 3 days. Compared with HT+placebo (0.9% sodium chloride), melatonin-treated infants demonstrated similar laboratory haematology, renal, liver, metabolic and coagulation parameters. Melatonin was not associated with increased seizure activity and MRI findings did not differ significantly between groups. At 18 months, melatonin-treated infants achieved higher cognitive scores and a trend to improved language scores. Melatonin was associated with reduced systemic proinflammatory cytokines.^32^ The authors concluded that melatonin was considered safe. The benefit on neurological outcomes remains inconclusive given the confounding effects (higher Sarnat Score in the placebo group) and small sample size of the study. Detailed PK data and adverse events (AEs) data were not reported.

Clinical studies of melatonin in babies with HIE meeting eligibility criteria for HT are now needed to assess safety and PKs. Based on reassuring safety data for intravenous melatonin dosing at 5 mg/kg, we plan for dose escalation starting at an intravenous loading dose of 5 mg/kg over 2 hours with five maintenance doses of 2.5 mg/kg over 2 hours every 12 hours from 24 hours. AEs and melatonin, BACs and, in *sentinel* babies, acetaldehyde levels will be measured to provide a comprehensive assessment of the recommended phase 2 dose (RP2D).

### Justification for the melatonin dosing regimen

The IMP will be administered intravenously as a loading dose within 6 hours of birth (T0) followed by five (half-dose) maintenance infusions from 24 hours (T0+24 hours) every 12 hours to cover the period of cooling and rewarming. All infusions will be given over 2 hours with close safety monitoring. Multiple doses of melatonin over this period are necessary given the evolving brain injury over hours and days after HI.

Given the lack of PK data in babies, dose escalation will proceed over four dose levels to achieve the target melatonin levels of 15–30 mg/L (based on the preclinical data), while assessing safety, and ensuring the BACs remain <0.25 g/L. Dose escalation will start at a dose of 5 mg/kg (plus five maintenance doses of 2.5 mg/kg), which has already been shown to be safe in this target population in a different formulation.^30^ Incremental increases of 5 mg/kg with subsequent dose levels are based on reassuring PK data for melatonin and BACs from simulations of a population PK model in newborn piglets. Each dose level chosen will be based on predictions from the concurrent human PK data as the ACUMEN Study progresses. Doses up to 30 mg/kg may be given based on the No Observed Adverse Events Level (NOAEL) in piglets.

While no absolute limit in BACs in medicines is stipulated by the UK Medicines and Healthcare products Regulatory Agency (MHRA) given the sparse safety evidence, we have set a limit of 0.25 g/L to align with previous recommendations.^33^ This balances the partial benefit of ethanol for HIE while limiting potential toxicity (BAC >1 g/L in rodents).^34^ In newborn piglets, this threshold does not lead to significant accumulation of acetaldehyde,^22^ the by-product of ethanol metabolism implicated in the systemic effect of ethanol toxicity. Blood acetaldehyde concentration will be measured in *sentinel* babies to ensure levels do not exceed 2.2 mg/L where mild, transient effects of facial flushing and tachycardia may present without other significant adverse effects.^35^,^38^

## Methods and analysis

### Study objectives

The overall aim of the ACUMEN Study is to assess the safety of four intravenous dose levels of melatonin in babies with moderate/severe HIE receiving HT.

The primary objectives are:

1. Safety profile assessment: to assess the safety profile of melatonin across all dose levels being studied based on the occurrence of a dose-limiting event (DLE).
2. The attainment of putative therapeutic plasma melatonin levels (15–30 mg/L) across dose levels being studied.
3. The attainment of putative BACs safety (<0.25 g/L) across dose levels being studied.
4. To identify the RP2D.

The secondary objectives are:

- PK model: To establish the PK profile of intravenous melatonin infusion in term infants with HIE undergoing HT.
- Feasibility of the neonatal neuroprotection trial network: To assess the feasibility of developing a neonatal neuroprotection trial network for future phase 2 RCTs.
- Recruitment feasibility: To assess the feasibility of recruiting participants within 6 hours of birth.

The exploratory objectives are:

- Biomarker discovery feasibility: To assess the feasibility of developing a standardised approach to exploratory blood biomarker discovery integrated with neuroprotection trials to identify early markers of HIE severity and treatment response.
- Dose-response assessment: To evaluate the dose-response curve for intravenous melatonin infusion in the target population.
- To develop a physiologically based PK model of melatonin and ethanol.

### Trial design

This multicentre, phase 1 safety study will recruit from level 3 neonatal intensive care units (NICUs) with experience in managing babies receiving HT for HIE across England, Scotland, Ireland and Australia. We aim to recruit 60 babies with moderate/severe HIE receiving HT starting August 2025, in two phases: (1) A 12-month dose escalation phase, to establish safety and identify the RP2D (24–39 babies), and (2) A 6-month cohort expansion phase, to affirm safety and PK of the RP2D (21–36 babies).

### Dose escalation procedure

**Figure 2:**
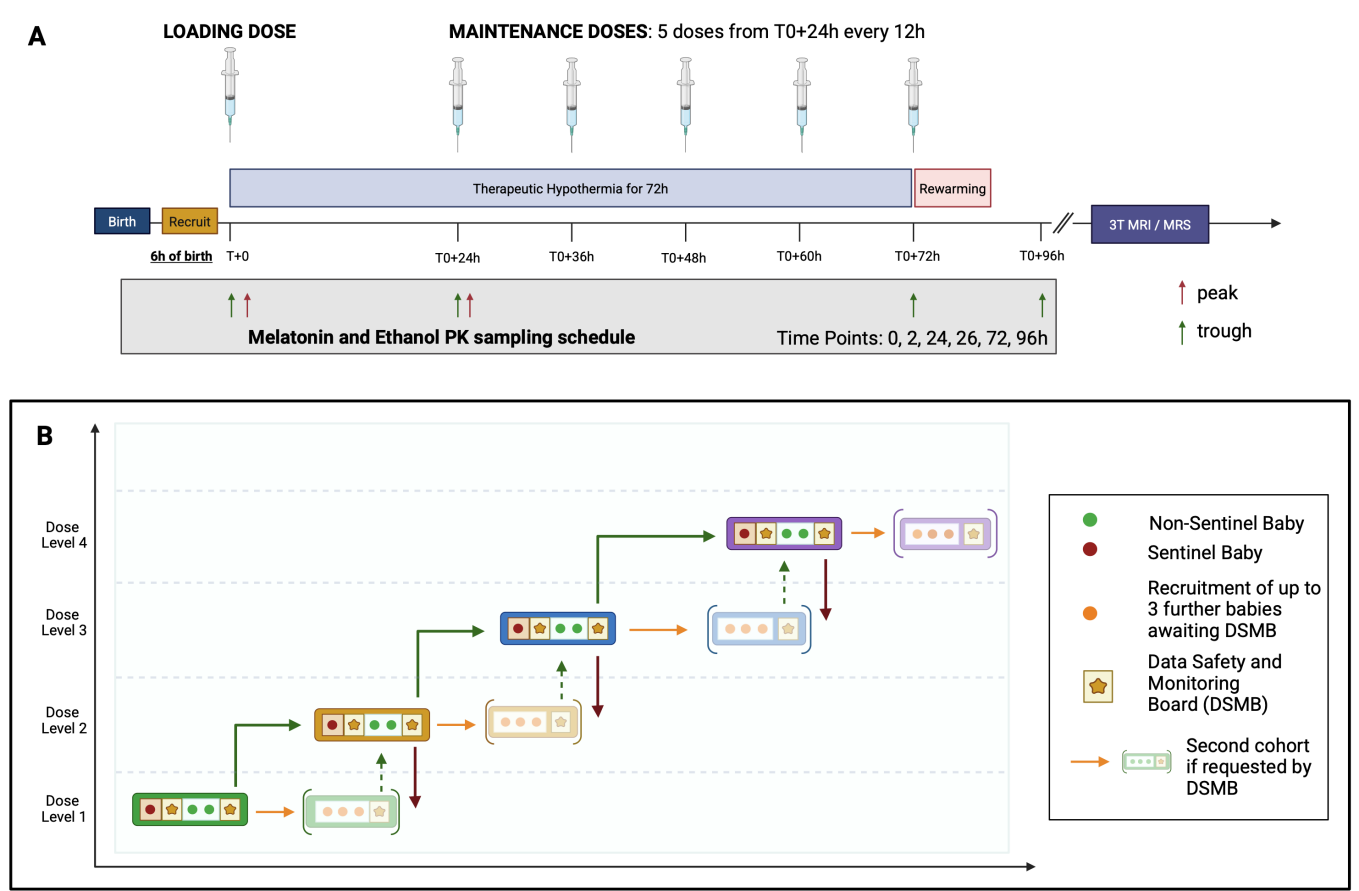
Melatonin dosing regimen and timing of pharmacokinetic (PK) blood sampling (**A**) and illustration of the principles of the dose escalation study design (**B**). Four dose levels will be assessed with a *sentinel* baby (red circle) at each dose level to ensure levels of melatonin (<33.5 mg/L), ethanol (<0.25 g/L) and acetaldehyde (<2.2 mg/L) are within safety thresholds after the loading dose. Subsequently, a further two babies at each dose level will be recruited prior to review of safety and PK data by the DSMB. Recommendations by the DSMB to the Trial Steering Committee to either (1) Dose escalate (2) Continue the same dose level for a further cohort of three babies, or (3) Reduce the dose (from dose level 2). The DSMB will also be called for review if there are any dose-limiting events. The figure is an illustration of one possible path of dose escalation in ACUMEN. Other paths, including with dose de-escalation or repeats of dose levels, are possible. Created in BioRender. Pang R (2025) https://BioRender.com/xlkdsdd. MRS, magnetic resonance spectroscopy.

The dose escalation phase (illustrated in figure 2) will assess up to four dose levels using the six potential doses listed in table 1. Participants will be allocated to a single dose level and overall, a minimum of three participants will be enrolled per dose level. AEs, based on the Neonatal Adverse Event Severity Score (NAESS)^39^ and blood samples over six time points, will be collected to measure melatonin and BACs as shown in figure 2.

**Table 1:** Melatonin doses under investigation based on the NOAEL of 30 mg/kg observed in piglets

Dose level	Loading dose at T0	5 maintenance doses (from T0+24 hours)
Dose 1	5 mg/kg	2.5 mg/kg
Dose 2	10 mg/kg	5 mg/kg
Dose 3	15 mg/kg	7.5 mg/kg
Dose 4	20 mg/kg	10 mg/kg
Dose 5	25 mg/kg	12.5 mg/kg
Dose 6	30 mg/kg	15 mg/kg

NOAEL, No Observed Adverse Events Level.

Dose level 1 will start with a loading dose of 5 mg/kg given within 6 hours of birth (T0) followed by five maintenance doses of 2.5 mg/kg from T0+24 hours every 12 hours. Blood melatonin levels and BACs will be measured over six time points based on PK modelling using piglet data.

The first baby recruited to each dose level will be a *sentinel* baby and subjected to the following safety precautions:

- They must have *moderate *HIE based on aEEG/EEG or Modified Sarnat Neurological Examination.
- In addition to plasma melatonin and BAC, acetaldehyde levels will be measured following the loading dose (at T0+2 hours) and data made available prior to subsequent maintenance dose to ensure the following PK safety limits are met:
  - Plasma melatonin <33.5 mg/L
  - Plasma acetaldehyde <2.2 mg/L
  - Blood alcohol concentration <0.25 g/L.
- If melatonin, BACs and acetaldehyde levels are not within the stated levels or not available, no further doses will be administered. In addition, further IMP administration to the *sentinel* baby must *not *continue if the *sentinel* baby experiences a DLE.

The clinical summary, safety and PK data will be reviewed by the Data Safety and Monitoring Board (DSMB) following 100 hours after last IMP administration (‘DLE safety window’) to determine whether the recruitment to the dose level may continue. Once safety has been confirmed in the *sentinel* baby, recruitment of subsequent babies may occur in parallel. Due to logistical constraints, only melatonin levels and BACs will be measured for the remaining participants at this dose level.

The DSMB will review the safety and PK data following the recruitment of the third baby in a cohort. The DSMB will recommend to the Trial Steering Committee (TSC) one of the following possible outcomes: (1) Recruit at the next dose level, (2) Continue recruitment at the current dose level, (3) Reduce dose to one of the previous dose levels (where relevant), or (4) Stop the trial. Dose escalation decisions will be based on both the concurrent accumulating safety (suspected DLEs) and PK data, and their predictions at the next dose level. For PK, the trial pharmacologist will develop a population-PK model to simulate the plasma melatonin levels and BACs at the next dose level. If PK modelling predicts that melatonin systemic exposure will be equal to or exceed the Cmax of 33.5 mg/L and Area under the serum concentration vs. time curve for 0-24h (AUC24) of 454.7 mg*h/L or the BAC safety threshold (<0.25 g/L), that dose level will not be opened to recruitment. For DLE predictions, a Bayesian Escalation with Overdose Control (EWOC) approach will be used. EWOC uses a statistical model to estimate the risk of DLE per dose level with the target level of DLEs set at 33% for this trial. While awaiting dose escalation decisions (from the second dose level onwards), and in the absence of DLEs in the first three babies, a maximum of three further babies can be recruited to the current dose level after approval from the DSMB. In the event of a DLE, or having recruited the three additional babies, backfilling^40^ to the previous dose level can occur while awaiting the dose escalation decision. Data acquired from additional babies will be included in a subsequent dose escalation report for review by the DSMB/TSC. Backfilling will be limited to a maximum of 15 babies in the dose escalation study.

The subsequent dose escalation will proceed to dose level 2 at a loading dose of 10 mg/kg and maintenance doses of 5 mg/kg. Subsequent doses for dose level 3 and dose level 4 will be determined by the safety and PK model prediction. Provided that dose levels 1 and 2 have not exceeded the maximum BAC safety threshold and no other dose-limiting toxicity is encountered, further escalation will be recommended. Using the parameters of a population PK model, dose escalation will be evaluated. The possible scenarios are:

- The model predicts the dose to achieve the melatonin target is lower than the dose to reach the dose-limiting BAC threshold. In this case, dose level 3 will aim to achieve the melatonin target.
- The model predicts the dose to achieve the melatonin target is equal to the dose to reach the dose-limiting ethanol target. In this case, dose level 3 will be set at one level below the predicted target dose.
- The model predicts the dose to achieve the melatonin target is greater than the dose to reach the dose-limiting ethanol target. In this case, dose level 3 will be set at the level predicted to be immediately below the predicted dose-limiting ethanol cut-off.

Following dose level 3, the PK model will be updated and dose level 4 set according to a dose level predicted to be immediately below the predicted dose-limiting BAC cut-off. At the end of the dose escalation phase, RP2D will be established.

### Cohort expansion study

The cohort expansion study will recruit a minimum of 21 and maximum of 36 babies to further assess the safety and PK of RP2D. The DSMB will convene after every 10 participants are enrolled to assess PK and the overall safety data.

It is anticipated that if the acetaldehyde data in the dose escalation study are reassuring, the expansion cohort will not include blood acetaldehyde levels as part of the PK blood analysis.

### Exploratory biomarker study

Exploratory blood marker studies are integral aspects to neonatal neuroprotection trials.^41^ Blood inflammatory cytokines,^42^,^44^ microRNA,^45^ neurospecific proteins^46 47^ and metabolomics^48 49^ have shown promise in predicting severity of HIE, 2-year neurodevelopmental outcomes and developing our understanding of the molecular pathways activated in babies with HIE. As brain injury is a temporal process, evolving with time, blood samples over several time points are necessary.

**Figure 3:**
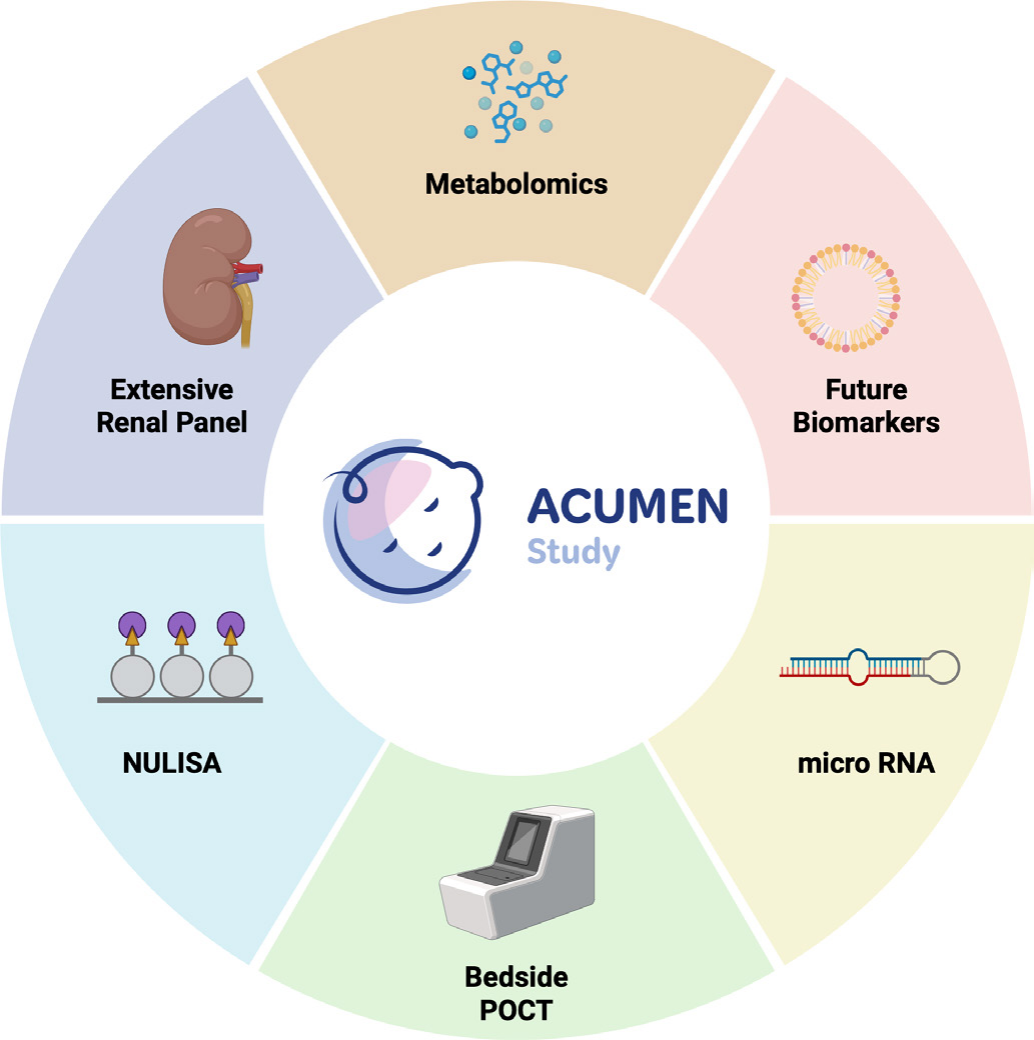
Exploratory Biomarker Study in the Acute High Dose Melatonin for Encephalopathy of the Newborn (ACUMEN) Study. The optional exploratory biomarker study aims to use the next generation of novel technology to identify promising biomarkers of hypoxic-ischaemic encephalopathy (HIE) severity. This includes validating rapid POCT UCH-L1 and GFAP test for HIE. Plasma will also be assessed using NUcleic acid Linked Immuno-Sandwich Assay (NULISA), a novel, proteomic liquid biopsy platform with high attomolar sensitivity and high multiplexing to profile over 120 disease markers, metabolomics, microRNA and assessment of renal injury. Created in BioRender. Pang R (2025) https://BioRender.com/ka7nocl.

The exploratory biomarker study is an optional parallel study aimed to use the next generation of novel technology to identify promising biomarkers of HIE severity. Blood samples at recruitment, T0+24 hours, T0+48 hours, T0+72 hours and T0+96 hours will be taken to assess rapid point-of-care test (POCT) Ubiquitin Carboxyl-Terminal Hydrolase L1 (UCH-L1) and Glial Fibrillary Acidic Protein (GFAP),^50^ NUcleic acid Linked Immuno-Sandwich Assay (NULISA),^51^ micro-RNA, metabolomics and an extensive renal function panel (see figure 3).

### Study intervention and outcomes

#### Eligibility criteria

To account for differences in cooling criteria across different neonatal units, the trial is pragmatic and will include babies eligible for HT according to local guidelines. Clinical data including blood gas, neurological examination and background aEEG/EEG activity will be collected for analysis.

1. Baby admitted to the NICU with moderate/severe HIE meeting eligibility criteria for HT (in accordance with local guidelines) and:
  1. Born at ≥36 completed weeks gestation.
  2. Clinically stable* at the time of IMP administration.
  3. Invasive blood pressure monitoring in situ prior to the administration of the IMP loading dose.
2. All participants will undergo a further assessment of HIE grade as determined by aEEG/EEG and/or a Modified Sarnat neurological examination prior to IMP administration.
  1. Sentinel Participant Criteria Only: must not meet the criteria for severe HIE.
3. Informed consent from parents/guardians/person with legal responsibility.

*Definition of Clinical Stability

Eligibility of the participant must be rechecked prior to every IMP administration given the varying clinical status of these infants. Stability will take the following into consideration:

- Well-placed central venous catheter or patent peripheral cannula in situ.
- Mean blood pressure (with or without inotropic support) must be greater than the fifth centile for gestation within 30 mins prior to IMP administration.
- Clinical or electrical seizures, if present, are controlled with antiseizure medications.
- Clinical observations within an acceptable range for an infant undergoing HT.
- No clinical stability concerns from the attending neonatologist.

#### Exclusion criteria

1. Baby >6 hours of age when IMP administered.
2. Initiation of IMP is unlikely to be administered within 6 hours of birth.
3. Infants born in very poor condition or judged too sick to be included (high risk of mortality) in an experimental first-in-human study, for example, infants that are requiring maximal intensive care therapy or in a condition considered to be life-limiting.
4. Postnatal hypoxic insult without any evidence of HIE at birth.
5. Birth weight less than the second centile for gestation on UK-WHO growth charts.
6. Congenital anomalies, that is, any major antenatally diagnosed congenital abnormalities such as congenital heart disease, suspected or known chromosomal abnormalities.
7. Head circumference less than second centile adjusted to sex of the baby on UK-WHO growth charts.
8. Infant is participating or intends to participate in another interventioanl study during the birth hospitalisation (not including oservational studies).
9. Parents/legal guardians unable to give consent due to leanring or other difficulties.

In the unlikely event of multiple births, if one baby has HIE, participation in the trial will be offered. If both/multiple babies have HIE, participation in the trial will not be offered.

### Recruitment and consent

The parent/legal guardian(s) of eligible participants will be identified by the clinical team on admission to the neonatal unit for cooling and asked for their willingness to participate in the trial. Informed consent will be obtained by a medically qualified investigator or authorised delegate of the study team. Given the time-critical nature of the trial and associated traumatic perinatal circumstances, a recruitment video and short patient information leaflet will be provided in addition to a more detailed patient information sheet. Verbal consent may be obtained initially, witnessed by an independent member of the clinical team, followed by written consent no later than 24 hours after birth. Separate consent will also be obtained for the optional biomarker study. Use of local language translation services to facilitate the consenting process in non-English-speaking parent/legal guardian(s) is permissive where the investigator is confident the discussion was accurately understood.

### Study intervention

The IMP, melatonin in ethanol 50 mg/mL solution for infusion, is a clear solution presented in 6 mL sterile amber vials, manufactured in accordance with GMP by Eurofins Proxy Laboratories under their manufacturing authorisation licence. Orphan drug designation has been obtained for HIE with the European Medicines Agency (EU/3/23/2757) and the US Food and Drug Administration (DRU-2022–8888). The solution is stored at 2°C to 8°C and requires dilution in 5% glucose to a final melatonin concentration of 2 mg/mL (3.8% v/v ethanol) for intravenous use. The IMP should be started as a 2-hour infusion within 6 hours of birth (T0) via a separate lumen of a well-placed central venous catheter or peripheral 24G cannula. Subsequently, five maintenance doses will be administered every 12 hours from T0+24 hours over 2 hours. Clinical stability (as stated in the eligibility criteria) must be assessed and confirmed prior to each IMP administration. Where the baby is not stable at assessment but subsequently becomes stable within 1 hour of the scheduled dosing event, the IMP may be delivered. Where a dose was missed due to clinical instability, the next IMP dose must be administered in accordance with the treatment schedule. No extra IMP doses outside the treatment schedule will be allowed.

### Criteria for discontinuing or modifying interventions

Any discontinuation or modification of doses will be in line with the trial design and in accordance with the dose escalation procedure confirmed by the DSMB and TSC. Reasons for discontinuing treatment include:

1. Serious Adverse Event (SAE) or “severe” AE, that is, grade 3 and above on the Neonatal Adverse Event Serverity Scale (NAESS)^39^ considered related to IMP (definition of suspected DLE)
2. PK exposure cap of melatonin (<33.5mg/L), ethanol (<0.25g/L) or acetaldehyde (<2.2mg/L) exceeded (this will be available concomitantly in the *sentinel* baby only). PK exposure cap of melatonin, ethanol or acetaldehyde predicted to exceed at the subsequent dose escalation.
3. Withdrawal of consent by parent/legal guardian(s).
4. A decision made by the treating clinician based on any change in the infant’s condition that justifies the discontinuation of treatment.

The analysis will be by intention to treat. Participants who discontinue protocol treatment, for any of the above reasons, should remain in the trial for the purpose of follow-up and data analysis. A blood sample will be collected at the time of termination and an additional sample 24 hours after the last IMP administration. These samples will be analysed for PK purposes to characterise the plasma levels of melatonin and ethanol.

### Concomitant care

Melatonin is metabolised by the hepatic cytochrome P450 CYP1A enzymes, primarily CYP1A2; therefore, interactions between melatonin and other active substances as a consequence of their effect on CYP1A enzymes are possible. Administration of CYP1A2 inhibitors (eg, allopurinol, ciprofloxacin, amiodarone) to ACUMEN participants should be avoided, where possible. The sponsor will collect a list of all concomitant medications given to participants on the concomitant medications log. The IMP treatment will be administered alongside standard neonatal neurocritical care for babies receiving HT for HIE. These include:

1. Continuous aEEG/EEG.
2. Cerebral near infrared spectroscopy (NIRS).
3. MRI and proton (^1^H) MRS on days 4–10 (preferred timing days 4–6)

### Amplitude-integrated electroencephalography /electroencephalography (aEEG/EEG)

Continuous aEEG/EEG will be initiated prior to IMP administration and continued for 24 hours after rewarming. For the ACUMEN Study, a full neonatal aEEG/EEG recording will be collected using the Stratus NICU EEG system (Stratus EEG software version 5.1.3). Time to recovery of aEEG/EEG background activity and sleep wave cycling accurately predicts adverse neurodevelopmental outcomes at 18 months (positive predictive value (PPV)=96.2%, negative predictive value (NPV)=88.92%, and PPV=88.5%, NPV=82.4%, respectively).^26^ In addition, seizure detection and prompt treatment is essential given that up to 50% of HIE infants develop seizures,^52^ with ~20% occurring during rewarming.^53 54^

### Cerebral NIRS

NIRS monitoring provides insight into regional tissue oxygen saturation (rSO_2_), indicating the balance between oxygen delivery to tissues and tissue oxygen utilisation at the bedside. The ACUMEN Trial will collect NIRS data over the period of cooling and rewarming using the Masimo Root with the O3 Regional Oximetry system. In HIE,^55^ elevated cerebral regional oxygen saturation (CrSO_2_) within 6 hours of birth,^56^ during cooling^57 58^ and rewarming,^58 59^ correlates with adverse neurological outcomes.

### MRI/magnetic resonance spectroscopy (MRS)

Conventional MRI provides information regarding the nature and severity of the insult, timing and prognosis. This is implied by the location and extent of injury and the signal characteristics on T1-weighted (T1W), T2-weighted (T2W) and diffusion-weighted MRI (dMRI). For optimal detection of evolving injury with dMRI, the scan should be acquired within the first 10 days after birth prior to pseudo-normalisation, with the optimal days of acquisition between days 4 and 6.^60 61^ Conventional MRI with dMRI predicts adverse neurodevelopmental outcomes at 2 years following HIE with a high degree of accuracy (Area under the Receiver Operating Characteristic (ROC) curve of 0.89 to 0.91).^62^ An imaging contract research organisation (CRO) (Bioxydyn) will assist with harmonisation of the multivendor 3T MRI scanners (Philips, Siemens and General Electric (GE)) using MRS and dMRI phantoms to ensure reproducibility and standardisation of MRS and dMRI. Data acquisition and analysis will meet regulatory expectations (International Council for Harmonisation (ICH) Good Clinical Practice (GCP)) with a controlled and auditable workflow.

Conventional MRI scans will be reported by the local radiological team and used for routine clinical management and counselling at each site. In addition, conventional MRI scans (including T1W, T2W, dMRI, susceptibility weighted imaging (SWI) and magnetic resonance venography (MRV) sequences) will be pseudonymised and sent to Bioxydyn from where they will be forwarded to two independent neonatal neurologists with extensive experience in assessing neonatal MRIs, who are members of the ACUMEN consortium. MRIs will be scored using the validated Rutherford^63^ and Weeke^62^ Scores. At this stage, the scores will be blinded, but the dose escalation level will be known. These scores will be used throughout the phase 1 study for safety monitoring and will be part of the data assessed by the DSMB at each dose level. At the end of the study, the MRIs will be batched for final radiological scoring, when the assessors will be blinded, including blinding to the IMP dose level administered. Incidental findings are likely to have been picked up by the site clinical radiologist as part of standard care. Unexpected MRI findings that could be related to the IMP will be reported to the DSMB.

The MRS Lac/NAA peak area ratio acquired from the left BGT voxel using a long echo time (TE 288 msec) has a high predictive accuracy with a sensitivity and specificity, respectively, of 100% and 97% for motor outcome, 90% and 97% for cognitive outcome, and 81% and 97% for language outcomes using a Lac/NAA threshold of 0.39.^28^ The Lac/NAA peak area ratio has been used as a surrogate outcome measure in a phase 2 imaging biomarker neuroprotection trial for HIE^7^ and validated against 2-year neurodevelopmental outcomes.^64^ The Lac/NAA is currently the best available, pragmatic predictor of 2-year neurodevelopmental outcomes.^28 29^ The use of a robust surrogate imaging biomarker such as MRS Lac/NAA has the potential to expedite clinical translation by evaluating treatment effects in a timely fashion with smaller sample sizes. Developing a strong infrastructure for a high standard of MRI/MRS harmonisation in the ACUMEN Study will be vital for the success of future phase 2 surrogate imaging biomarker neuroprotection trials.

dMRI data will be processed to derive peak width of skeletonised mean diffusivity and fractional anisotropy. These are image markers of white matter microstructure based on diffusion tensor imaging, skeletonisation of white matter tracts and histogram analysis.^65^ The processing pipeline has been optimised for neonatal dMRI data^66^ and is suitable for analysing data acquired at multiple sites.^67^

### Outcomes

The primary outcome, to identify the RP2D, is based on:

- Safety: The highest dose level that has an estimated probability of DLE closest but below the target DLE level of no greater than 33%. The totality of the safety data will also be considered.
- PK: Achieving and maintaining putative melatonin plasma levels of at least 15–30 mg/L and ensuring BAC remains <0.25 g/L. The PKPD (dose-response curve stratified by severity of HIE) model may also be considered.

### Dose-limiting events

Suspected DLEs are defined as any events listed below considered *related* to the IMP by the attending neonatologist and site investigator:

- Severe AE defined by any single event that is grade 3 and above on the NAESS^39^
- Serious AE (SAE)

In the event that a suspected DLE occurs, the sponsor will be notified within 24 hours and the event will be reviewed by an independent clinical reviewer. The DSMB will review the data to confirm the DLE and advise on whether to continue recruitment at the same dose level or recruit to a lower dose level. If a suspected DLE occurs in a *sentinel* baby, at the discretion of the DSMB and TSC, a second *sentinel* baby may be requested at the same dose level; however, if a further suspected DLE occurs in this second *sentinel* baby, the dose will be considered unsafe, and no further dosing will occur at this dose level or higher.

### Participant timeline

The infusion of the loading dose of IMP will be given as soon as possible after birth (T0), once arterial and venous access has been obtained and the participant is deemed clinically stable. IMP must be administered no later than 6 hours after birth with five subsequent maintenance doses given at T0+24 hours, T0+36 hours, T0+48 hours, T0+60 hours and T0+72 hours (±1 hour) via an individual lumen of a well-placed central venous catheter or peripheral venous cannula. Screening for clinical stability, assessments and investigations will be performed in accordance with the schedule of events summarised in table 2 with a detailed schedule outlined in the supplementary materials (online supplemental table S3). Clinical stability must be confirmed within 30 mins prior to each scheduled IMP administration. Further clinical safety data during the infusion of the IMP will be collected as outlined in table 3. This includes intensive vital signs (including invasive blood pressure) monitoring every 15 mins during the loading dose, liberalising to every hour during the maintenance doses. In the event that invasive blood pressure monitoring is lost after T0+48 hours, IMP administration may continue provided the baby is not on inotropic support and has not experienced a DLE.

**Table 2:** Summary schedule of events

Schedule of events	Screening and T0	T0+ 24 hours (±1 hour)	T0+ 36 hours (±1 hour)	T0+ 48 hours (±1 hour)	T0+ 60 hours (±1 hour)	T0+ 72 hours (±1 hour)	T0+day 7 (±1 day)	Hospital discharge
Informed consent	X							
Eligibility	X							
Clinical stability	X	X	X	X	X	X		
Baby demographics	X							
Maternal characteristics	X							
Pregnancy and delivery history	X							
Placental histology	X							
Physical examination	X							
Therapeutic hypothermia	X	X	X	X	X	X		
IMP administration	X	X	X	X	X	X		
Cranial ultrasound with resistance index	X	X		X		X		
aEEG/EEG	X	X	X	X	X	X		
NIRS	X	X	X	X	X	X		
Modified Sarnat neurological examination	X	X		X		X	X	X
Concomitant medications	X	X	X	X	X	X	X	X
Review of AE and SAEs	X	X	X	X	X	X	X	X
Cord/blood gas pH/base deficit	X							
Biochemistry (renal, liver profile, CRP, troponin, CK, LDH)	X	X		X		X		
Blood culture	X							
Blood gas	X	X		X		X		
Full blood count	X	X		X		X		
Clotting including fibrinogen	X							
MRI/MRS neuroimaging								
Hammersmith Neonatal Neurological Examination (HNNE)								X
Hearing screen								X
Discharge characteristics								X
General movement assessment								X

The full detailed schedule is included in the supplementary materials (online supplemental table S3).

AE, adverse event; aEEG/EEG, amplitude-integrated electroencephalography; CK, creatine kinase; CRP, C-reactive protein; IMP, investigational medicinal product; LDH, lactate dehydrogenase; MRS, magnetic resonance spectroscopy; NIRS, near infrared spectroscopy; SAE, serious adverse event.

**Table 3:** Vital signs monitoring during IMP administration

	During the loading dose (initiation of infusion = L0)		During the maintenance dose (initiation of each infusion = M0)	
	Every 15 mins during the 2-hour loading dose infusion (L0 to L0+2 hours)	At L0+6 hours, 12 hours, 18 hours and 24 hours (L0+2 hours to L0+24 hours)	Every 1 hour during the maintenance doses (M0 to M0+2 hours)	At M0+6 hours and M0+12 hours (M0+6 hours to M0+12 hours)
Mean arterial blood pressure*	X	X	X	X
Core temperature	X	X	X	X
Ventilation (mode, FiO_2_ requirement)†	x	X	X	X
Cerebral NIRS rSaO_2_	X	X		
Heart rate	X	X	X	X

* In the event that invasive arterial access is lost after 48 hours from the start of the loading dose (L0+48 hours), provided there is no DLE and the baby is not on inotropic support, melatonin may be continued and non-invasive blood pressure monitoring recorded.

† Ventilation data will be recorded every 30 mins during the loading dose.

DLE, dose-limiting event; FiO2, fraction of inspired oxygen ; IMP, investigational medicinal product; NIRS, near infrared spectroscopy; rSaO2, regional oxygen saturation.

Blood (PK) samples will be obtained (0.5 mL in sodium fluoride/potassium oxalate) at T0, T0+2 hours, T0+24 hours, T0+26 hours, T0+48 hours, T0+96 hours to measure peak and trough melatonin and BAC levels. Parents, guardians and those legally responsible for the participant may also give consent to the exploratory biomarker study outlined in figure 3 with the schedule of blood sampling shown in table 4. Where available, all samples will be obtained from the arterial line; however, if this is no longer available after T0+48 hours, the PK sample must be obtained via venepuncture. The exploratory biomarker sample may be obtained opportunistically with routine bloods. Where the participant is anaemic (haemoglobin (Hb) <100 g/L), the exploratory biomarker sample must not be taken. Standard-of-care blood results (full blood count, renal and liver profiles, coagulation screen, blood culture, CRP) will also be collected as part of the trial.

**Table 4:** Exploratory blood biomarker sample schedule

Sampled	Screening /baseline	T0+24 hours (+12 hours)	T0+48 hours (+12 hours)	T0+72 hours (+12 hours)	T0+96 hours (+12 hours)
Blood plasma sample (1 mL in EDTA)	X	X	X	X	X
Dried blood spot for microRNA (80 uL of whole blood)	X	X	X	X	X
Point-of-care GFAP/UCH-L1 (10 uL of whole blood)	X	X	X	X	X

GFAP, glial fibrillary acid protein; UCH-L1, ubiquitin carboxyl-terminal hydrolase L1.

Following completion of AE monitoring and neonatal intensive care, babies will be discharged with outpatient follow-up at 3 months. Prior to discharge, neurological assessments including the Hammersmith Neonatal Neurology Examination and video for General Movement Assessment will be performed as part of standard care as shown in table 1. At follow-up, the Ages and Stages Questionnaire, the Hammersmith Infant Neurological Examination and a further General Movement Assessment video will be obtained.

### Data collection and management

Participants meeting the eligibility criteria for the ACUMEN Trial will be enrolled via the Sealed Envelope online system, which will allocate participants to an individualised participant identification number (PIN) for pseudo-anonymisation of research data. During the dose escalation phase, assignment to the current dose level will be managed via the Sealed Envelope system in accordance with the dosing schedule approved by the TSC. Clinical data from participant records will be collected as illustrated in the schedule of events (see table 2) under the PIN into the password-protected study database (OpenClinica). MRI/MRS Digital Imaging and Communications in Medicine (DICOM) data will be transferred to VoxelFlow software, anonymised and reviewed by two independent experts in perinatal brain MRI interpretation. The aEEG/EEG recording will be uploaded to the Stratus EEG system software for review by a neurophysiologist with expertise in neonatal neurology. Where available, the NIRS trace will be transferred to the UCL Data Safe Haven for review. Laboratory data (including PK) will be provided directly to the sponsor. All PK final reports will be entered into OpenClinica linked to the PIN.

All data will be handled in accordance with the Data Protection Act 2018 and the EU General Data Protection Regulation (GDPR) 2016. The data are stored on secure, GDPR-compliant, cloud-based servers held within the UK and EU. Any transfer of documentation containing personally identifying data between the site and the coordinating centre will be subject to AES-256 industry-standard encryption.

### Analysis of biological samples

The plasma levels of melatonin, ethanol and acetaldehyde will be measured by a central Good Laboratory Practice (GLP) laboratory (Analytical Services International (ASI), London); plasma melatonin levels will be measured using liquid chromatography-tandem mass spectrometry (LC-MS/MS), ethanol and acetaldehyde levels will be measured using headspace gas chromatography-flame ionisation detection (Headspace GC-FID).

Whole blood UCH-L1 and GFAP concentration will be measured in real time at the cot side using the Abbott Traumatic Brain Injury cartridge and Alinity POCT device according to the manufacturer’s instructions. All plasma and dried blood spots will be processed at the local sites, stored at −80°C and transferred to the UCL Neonatal Neuroprotection Research Group Laboratory for biobanking at the end of the study. Plasma protein concentrations will be measured using the Alamar NULISA Central Nervous System Disease panel array by the UCL Dementia Research Institute Biomarker Factory. Qiagen will measure an array of microRNA markers previously shown to have predictive potential in HIE.^45^ Plasma metabolomics will be measured using non-targeted rapid liquid chromatography mass spectrometry analysis by Sapient Bioanalytics. The remaining samples will be stored for future exploratory biomarker studies.

### Sample size and statistical analysis

This is a dose-finding, safety study. The sample size of 60 participants is based on the collection of sufficient safety data to estimate the risk of DLE and PK profile at each dose level, plus a cohort expansion cohort to assess recruitment feasibility within the time targets of the trial.

The escalation will proceed according to the principles of the Bayesian Escalation with Overdose Control (EWOC) approach. The maximum tolerated dose (MTD) is defined to be the dose level of melatonin that results in a probability θ=33% that a DLE will occur in the period between the initiation of IMP treatment and 100 hours after the final administration of IMP, that is, P (DLT | dose = MTD) = 33%. The expected proportion of babies treated at doses above the MTD will be controlled at a maximum of 25% (EWOC feasibility bound α, kept constant). We use the classical specification of EWOC with a two-parameter logistic curve between dose and probability of DLE .^68^ The two parameters of the re-parameterised model, γ (the MTD) and ρ (the probability of DLT at the dose level 1), are given Uniform (ie, β (1,1)) prior distributions. For γ, the range is from the lowest dose to the highest dose. For ρ the range is from 0% to θ=33%.

We will use cohorts of three patients during the dose escalation part of the study. The operating characteristics of the dose-finding design are based on DLE occurrence for three key scenarios, based on 1000 simulations of the study per scenario (see table 5) for 24 dose-finding babies in cohorts of three. The first scenario is such that the MTD is expected to be the fourth dose level (ie, γ=4) and ρ=0.1. The second scenario is one where the fourth dose level is too toxic (P (DLE | dose=4) = 51%) and the MTD is the third one. In the third scenario, the probability of DLE is generally high (ρ=0.15, P (DLE | dose=4) = 79%), and the MTD is the second dose level.

**Table 5:** Operating characteristics of the Bayesian Escalation with Overdose Control (EWOC) approach

	First dose level	Second dose level	Third dose level	Fourth dose level
DLE Curve				
Scenario 1; DLE probability	0.10	0.15	0.23	0.33 (MTD)
Scenario 2; DLE probability	0.10	0.19	0.33 (MTD)	0.50
Scenario 3; DLE probability	0.15	0.33 (MTD)	0.58	0.79
Recommendation percentages (1000 repetitions). The proportions are the percentage of the simulations that recommended each dose level. For example, in scenario 1, dose level 3 was recommended in 69% of the simulated trials.				
Scenario 1; recommendation (%)	0.0	28	69	3
Scenario 2; recommendation (%)	2	50	48	0
Scenario 3; recommendation (%)	10	89	1	0

DLE, dose-limiting event; MTD, maximum tolerated dose.

### Interim review of DLEs and dose escalation decisions

Interim review of the safety and PK data will be performed as follows: (1) After each *sentinel* baby (2) After recruitment of a cohort of three infants per dose level for dose escalation decisions and (3) After a suspected DLE. The purpose of the interim review will be to affirm safety and PK. Following completion of the DLE safety window (100 hours after last IMP administration), the safety and PK data will be source-verified by the sponsor and presented to the DSMB for review. The DSMB will review the data and advise the TSC on whether dosing at the current level may continue (for example after a *sentinel* baby or DLE event) or whether a dose escalation to the next dose level is recommended (after a cohort of three babies at a dose level). For the *sentinel* baby at each dose level, no other baby may be recruited until the interim safety and PK data have been reviewed by the DSMB/TSC. For the purpose of dose escalation decisions, the trial statistician and pharmacologist will provide updated estimates of dose-limiting toxicity and PK predictions at the next dose level. In the cohort expansion phase, the safety and PK data will be reviewed after every 10 babies. No interim analysis will be performed for the secondary and exploratory outcomes.

The study will be terminated if any of the following stopping rules are satisfied:

- Emergence of new therapies for HIE of greater benefit to this population.
- The DSMB recommends terminating the trial for safety reasons or if any evidence suggests that the risk-benefit ratio of the clinical trial is unfavourable.
- AEs: The incidence or severity of AEs in this study indicates a potential risk to patients taking part in the trial. Any isolated NAESS grade 5 AE (death) considered related to the IMP will result in immediate stopping of recruitment at the current dose level. No further participants will be recruited to that dose level until review by the DSMB and TSC.
- PK: If the observed or predicted melatonin, ethanol, or acetaldehyde levels exceed their respective Cmax cap in the *sentinel* baby, further dosing at this dose level must not proceed until DSMB/TSC review.
- Significant protocol deviation, lack of compliance or cooperation on the part of an investigator, which endangers the safety of the participants or the validity of the trial.

### Study oversight and monitoring

The UCL Comprehensive Clinical Trials Unit (CCTU) is the global sponsor for the study and is responsible for the overall trial oversight across all participating countries. UCL CCTU also acts as the sponsor for sites in the UK and Ireland. In Australia, the Flinders University Clinical Trials Unit will undertake sponsor duties at the national level under delegated authority, with UCL CCTU maintaining global trial oversight.

A Trial Management Group (TMG), comprising the Chief Investigator, CCTU, Patient and Public Involvement representative and other lead investigators, will be responsible for the design, coordination and strategic management of the trial. The TSC is an independent group providing trial oversight to safeguard the interest of the trial participants. The TSC, through its independent chair, will provide overall supervision and ultimately decide on the continuation of the trial. The DSMB is responsible for safeguarding the interests of the trial participants and monitoring the data for the trial. They are the only oversight body that has access to the individual participants’ data. Through their appointed chair, the DSMB will make recommendations to the TSC on whether the trial should continue as planned. The DSMB and TSC will independently meet after each *sentinel* baby and after every cohort of three babies for dose escalation decisions. In the situation where the TSC disagrees with the DSMB’s recommendation, a meeting will be held between the TSC and the DSMB to come to a shared decision. If a consensus is not made, then advice from the MHRA will be required.

UCL CCTU will implement a structured communication process to ensure participant safety and consistent site-level actions. Formal electronic notification of all sites will occur during sponsor working hours. In the event of any safety concerns, an urgent review by the DSMB may be convened. Where necessary, recruitment may be restricted via Sealed Envelope to prevent further dose allocation, and all sites will be formally notified by the sponsor.

Clinical trial monitoring will be conducted by the trial team at UCL CCTU in accordance with a predefined Quality Management and Monitoring Plan overseen by the UCL CCTU Quality Management Group. The monitoring strategy will involve 100% source data verification across all dose levels through on-site and remote visits, and central monitoring. Activities will include verification of eligibility, consent, IMP administration, safety assessments and reporting of AEs. Sites will undergo on-site monitoring following recruitment of a *sentinel* participant and again after the third participant at a given dose level. This will ensure all data are fully source-verified prior to submission to the DSMB and TSC for dose escalation decisions.

### Participant and public involvement

A parent-patient representative from Hope for HIE, a parent-patient advocacy group for HIE, is included within the TSC and TMG. The patient representative has been involved from the early planning stages of the trial. Participant-facing materials have been reviewed and suggestions incorporated to ensure clear communication of the trial risks and benefits.

### AEs reporting

All AEs related to IMP administration must be reported to the sponsor. All SAEs and severe AEs (defined as grade 3 and above on the NAESS), considered related to the IMP, must be reported within 24 hours of the event. Non-serious AEs related to the IMP must be reported within 7 days.

The reporting window for suspected DLEs extends to 100 hours after the final IMP administration, accounting for the expected five half-lives of the drug. Reporting of all IMP-related and unrelated SAEs must occur within 24 hours until 10 days after birth to account for the period of normalisation after neonatal intensive care. After 10 days, all SAEs related to IMP must continue to be reported within 24 hours, whereas unrelated SAEs will be reported to the sponsor within 7 days.

We anticipate that non-IMP-related AEs are common observations in this patient population; therefore, grade 1 and grade 2 (non-serious) events do not require reporting to the sponsor. These should be recorded in the participant’s medical record so that the sponsor can retrospectively collect these data if required. All serious non-IMP-related events (grade 3 or grade 4 on the NAESS)^39^ must be recorded in the participant’s AEs log and reported to the sponsor within 24 hours. This is with the exception of non-IMP-related events on the sponsor reporting exemption list (see online supplemental table S2) which are common complications of HIE. These exempt events will be recorded on the participant’s AEs log. If these events worsen or are more severe than expected, reporting to the sponsor within 24 hours is required.

Similar to IMP-related events, all non-IMP-related SAEs are reportable within 24 hours for the first 10 days after birth. After 10 days, non-IMP-related SAEs should be reported to the sponsor within 7 days. Monitoring and reporting of notifiable AEs will continue until participants reach 90 days from birth. Thereafter, active monitoring for AEs will not take place, but the sponsor must be notified of any serious adverse reactions until trial closure. No formal frequency of audit for this study will be performed, but the study will be overseen by the sponsor. An independent clinical review of all SAEs will be undertaken by the sponsor. All events classified as a Suspected Unexpected Serious Adverse Reactions will be onward reported by the sponsor to the regulatory authorities and ethics committees in accordance with applicable regulations.

The expectedness of events will be determined using the Reference Safety Information section of the approved Investigator’s Brochure.

### Significance of the study

More than two decades since the introduction of HT for moderate/severe HIE, many infants still face lifelong disabilities, profoundly affecting their lives, as well as the well-being of their families and the broader community. Importantly, cooling remains the only effective therapy available as adjunct therapies including xenon and erythropoietin have failed to show treatment benefit. Safe and effective adjunct therapies are urgently needed to complement cooling for moderate/severe HIE.

While there are no established guidelines to support the readiness of therapies for clinical translation in HIE, melatonin has demonstrated robust efficacy in both small and large animal models across multiple laboratories,^18^ fulfilling the Stroke Therapy Academic Industry Roundtable criteria applied in adult stroke research.^19^ The ACUMEN Study is a first-in-human phase I trial to establish the safe dose of a novel GMP melatonin in ethanol formulation for intravenous use to augment cooling for moderate/severe HIE. The development of highly concentrated melatonin for intravenous use is challenging given its poor solubility in aqueous solution. This study leverages the partial neuroprotective effects of ethanol, included as an adjunct excipient. Drawing on our experience in the UCL Neonatal Neuroprotection Research Group, we have established the safety, efficacy and PK of melatonin for HIE, and developed and refined a clinically relevant dosing regimen leading to the rationale and design of this trial. While a previous study of intravenous melatonin using alternative excipients has been reported,^30^ this first-in-human study of our novel melatonin formulation will comprehensively assess safety and PK through dose escalation. We will build on the safe dose of 5 mg/kg established by Jerez-Calero in the alternative formulation as the starting dose. The safety assessment uses the established NAESS developed by an international neonatal consortium to facilitate the conduct of neonatal clinical trials.^39 69^ The study will centralise PK analysis to an experienced GLP laboratory who have developed methodology to minimise blood volume requirements for the trial. Additionally, this study will comprehensively monitor BAC levels, which are rarely measured despite the presence of ethanol excipients in drugs administered to neonates.

In parallel to the primary objective to establish the R2PD dose of melatonin based on safety and PK, we aim to establish a neuroprotection trials network with optimised neurocritical care, including aEEG/EEG monitoring, NIRS and MRI/MRS. Using an imaging CRO, we aim to harmonise the 3T MRI systems across the network to ensure reproducible and standardised MRI/MRS acquisition. We will focus on the quantification of the MRS BGT Lac/NAA in this population due to its predictive power as a surrogate early read-out outcome biomarker to expedite clinical translation in future phase 2 RCTs. An optional blood biomarker study is integrated into this study to further develop bedside tools over the full time course of cooling and rewarming to accurately identify the babies with HIE at the greatest risk of adverse outcomes and therefore benefit most from further neuroprotective interventions.

### Dissemination and ethics

The study will be conducted in accordance with Good Clinical Practice. All results and analysis from the study will be published in peer-reviewed journals and presented at international conferences. A summary of the results will be submitted to the clinical trial registries in accordance with the participating countries’ requirements. A lay summary of the trial results will be disseminated in accordance with ethical and regulatory requirements in each participating country. This includes submission to public trial registries where applicable and sharing results in plain language with parent/legal guardian(s) of participants. This manuscript is based on the UK V.3.0 of the ACUMEN protocol (dated 8 May 2025). Separate protocol versions will be implemented in Ireland and Australia based on the UK protocol V3.0. This study has been approved by the UK National Health Service Health Research Authority and London Central Research Ethics Committee (REC reference 25/LO/0170). Clinical trial authorisation from the MHRA (CTA 11144/0003/001–0001) has been obtained in accordance with The Medicines for Human Use (Clinical Trials) Regulations 2004. Clinical trial authorisation from the Health Products Regulatory Authority under the Clinical Trials Regulation (EU) 536/2014 (Ireland) and from the Therapeutic Goods Administration under the Therapeutic Goods Regulations 1990 (Australia) is in progress at the time of publication.

Amendments to trial protocol, participant-facing materials or essential trial documentation will be submitted for review and approval to the relevant REC and competent authorities in each participating country, as applicable. The sponsor delegate (UCL CCTU) will coordinate communication of approved amendments to the study sites for local implementation.

## Supplementary material

10.1136/bmjopen-2025-107083 online supplemental file 1
