# Supplementary material for: Acute High Dose Melatonin for Encephalopathy of the Newborn (ACUMEN) Study: a protocol for a multicentre phase 1 safety trial of melatonin to augment therapeutic hypothermia for moderate/severe hypoxic ischaemic encephalopathy
Source: BMJ Open. 2025 Aug 22;15(8):e107083. doi: 10.1136/bmjopen-2025-107083 (PMC12374684; doi:10.1136/bmjopen-2025-107083)
Supplement: online supplemental file 1 [file bmjopen-15-8-s001.docx]

**Supplementary Material**

**ACUMEN Consortium Members**

***Members of the ACUMEN Trial Team***

| **Name** | **Affiliation** | **Role and responsibilities** |
| --- | --- | --- |
| Prof Nikki Robertson | UCL, London | Chief Investigator |
| Dr Raymand Pang | UCL, London | Researcher Co-Investigator |
| Ms Anvi Wadke | UCL CCTU, London | Clinical Project Manager |
| Dr Hakim-Moulay Dehbi | UCL CCTU, London | Trial Statistician |
| Ms Alyson MacNeil | UCL CCTU, London | Trial Manager |
| Mr Yusuf Jaami | UCL JRO, London | Regulatory Manager (Pharmaceuticals) |
| Dr Pamela Tranter | UCL TRO, London | Group Head, Translational Research Office |
| Alexa King | UCL CCTU, London | Data Manager |

***Members of the Trial Management Group***

| **Name** | **Affiliation** | **Role and responsibilities** |
| --- | --- | --- |
| Prof Nikki Robertson | UCL, London | Chief Investigator (CI)  TMG Chair |
| Dr Raymand Pang | UCL, London | Researcher Co-Investigator - TMG Deputy Chair |
| Dr Ajit Mahaveer | St Mary’s Hospital, Manchester | Principal Investigator (PI) |
| Dr Giles Kendall | University College London Hospital, London | Principal Investigator (PI) |
| Dr Julie-Clare Becher | Royal Infirmary of Edinburgh, Edinburgh | Principal Investigator (PI) |
| Prof Eleanor Molloy | Coombe Hospital, Dublin | Principal Investigator (PI) |
| Prof Adrienne Foran | Rotunda Hospital, Dublin | Principal Investigator (PI) |
| Prof Brian Walsh | Cork University Hospital, Cork | Principal Investigator (PI) |
| Prof Rod Hunt | Monash Children’s Hospital, Victoria | Principal Investigator (PI) |
| Dr Kathryn Martinello | Flinders Medical Centre, and Flinders University, Adelaide | Principal Investigator (PI) (Australian PI) |
| Prof Divyen Shah | Royal London Hospital, London | Principal Investigator (PI) |
| Dr Hakim-Moulay Dehbi | UCL CCTU, London | Co-Investigator/Head of Statistics |
| Dr Pamela Tranter | UCL TRO, London | Head of Translational Research Office |
| Anvi Wadke | UCL CCTU, London | Clinical Project Manager |
| Yusuf Jaami | UCL JRO, London | Regulatory Manager (Pharmaceuticals) |
| Alyson MacNeil | UCL CCTU, London | Trial Manager |
| Gina Abraham | Hope for HIE, USA | PPI Representative |
| Prof Subhabrata Mitra | University College London, London | Professor of Neonatal Medicine /Co-Investigator |
| Prof James Boardman | University of Edinburgh, Edinburgh | Professor of Neonatal Medicine/Co-Investigator |
| Prof Geraldine Boylan | University College Cork (UCC), Cork | Professor of Neonatal Physiology/Co-Investigator |
| Alexa King | UCL CCTU, London | Data Manager |
| Prof Deirdre Murray | University College Cork (UCC), Cork | Professor of Paediatrics/Co-Investigator |
| Dr Balamurugan Palanisami | Liverpool Women's Hospital, Liverpool | Principal Investigator (PI) |

**MRI/MRS Protocol**

MRI scans will be conducted using 3.0 Tesla scanners following harmonised imaging protocols.

In brief, the imaging protocol include:

- Coronal T1-weighted MRI with isotropic resolution (1×1×1 mm),
- Axial T2-weighted MRI (0.5×0.5×2 mm),
- Proton MR Spectroscopy (MRS),
- Diffusion-weighted MRI (DW-MRI) with 3 directions (b-value = 750),
- Susceptibility-weighted imaging (SWI),
- Venography,
- Isometric T2-weighted imaging (1×1×1 mm) used as a template for diffusion tensor imaging (DTI) analysis,
- DTI with 64 diffusion directions b-value = 750, and additional b0 volumes acquired with reversed phase encoding.

Proton MRS will be performed using a single voxel spectroscopy technique (TR = 2000 ms, TE = 288 ms) with a voxel size of 1.5×1.5×1.5 cm placed in the left thalamus. The acquisition time will be approximately 5 minutes. When feasible, a second MRS acquisition will be performed using a shorter TE of 35 ms to enable comparison of high and low TE performance for lactate detection.

**Supplementary Tables**

**Table S1:** Summary of preclinical data in newborn piglets following HI

| **Study** | | **Dose and formulation** | **PK Melatonin** | | **Excipient** | **Efficacy** | **Toxicology** |
| --- | --- | --- | --- | --- | --- | --- | --- |
|  |  |  | **Average Cmax (range)** | **AUC_24_** |  |  |  |
| 1 | **Robertson et al., Brain 2013**  Newborn Piglets <48h old with Hypoxia-ischaemia and cooling  N= 17 | **30 mg/kg/24h** started at 10 mins, infused over 6h - 2 doses (48h study)  Cooling 2-26h | 21 mg/L  (14.8 to 30.7)  within 3-6h of HI | **347.8 mg/L*h** | **Ethanol**  **2.5% v/v** | Significant brain protection with 3 outcome markers 1.EEG, 2.MR spectroscopy, 3. Widespread reduced cell death | No physiological or biochemical change with melatonin. No change in BP.  No hypoglycaemia (eth) |
| 2 | **Robertson et al., Neurobiol of Dis 2019**  Newborn Piglets <48h old with Hypoxia-ischaemia and cooling  N=30 | **5 mg/kg/24h** melatonin for 2 doses started at 2h, infused over 6h | 3.9 mg/L  (2.4 to 6.0) at 12h after HI | **66.3 mg/L*h** | sulfo-butylether beta-cyclodextrin | No protection | No physiological or biochemical change with melatonin. No change in BP. |
|  |  | **15 mg/kg/24h** for 2 doses started at **2h**, infused over **6h**  Cooling 2-26h | 16.8 mg/L  (6.2 to 25.0) at 8h after HI  (outside window of opportunity) | **231.7 mg/L*h** |  | No protection on EEG or MR spectroscopy, localised protection only of sensori-motor cortex |  |
| **3** | **Pang et al., Brain Comm 2021**  Newborn Piglets <48h old with Hypoxia-ischaemia and cooling  N=49 | **20 mg/kg/24h** melatonin for 3 doses started at **1h** and infused over **2h**  Cooling 1-13h | 27.8 mg/L  (19.5 – 36.2)  at 3h after HI | **411.9 mg/L*h** | sulfo-butylether beta-cyclodextrin | Significant brain protection with 1. EEG, 2. MR spectroscopy; 3. Localized protection only of sensorimotor cortex | No physiological or biochemical change with melatonin. No change in BP. |
| **4** | **Robertson et al., Scientific Reports 2020**  Newborn Piglets <48h old with Hypoxia-ischaemia and cooling | **18 mg/kg/24h** melatonin for 3 doses started at 1h and infused over 2h  Cooling 1-13h | 18.8 mg/L at 3h after HI | **287.9 mg/L*h** | **Ethanol:**  Partial protection with ethanol alone - on EEG recovery, MRS, cell death, oligodendrocyte survival | Significant brain protection 1.EEG, 2.MRS (1H and 31P), 3. Cell death reduced, oligo survival increased | No physiological biochemical change with melatonin (no change in BP)  No hypoglycaemia (eth) |
| **5** | **Pang and Robertson 2024**  Newborn Piglets <48h old with Inflammation-amplified  Hypoxia-ischaemia | **20 mg/kg** melatonin at 1h infused over 2h  then **10 mg/kg** melatonin 12hrly from 24h for 4 doses  No cooling | Loading dose:  33.5 mg/L  (22.5 to 46.4) by 3h  Maintenance doses:  30 mg/L (18.9 to 44.1) | **454.7 mg/L*h** | **Ethanol**  **PK: Loading** 0.32g/kg eth over 2h**:**  **BAC:** 0.18 +/- 0.02g/L  **Maintenance 12 hourly** 0.16g/kg over 2h  **BAC:** 0.049 +/- 0.02g/L | Significant brain protection with 1.EEG, 2.MR spectroscopy (1H and 31P), 3. Histology – increased NeuN survival | No physiological or biochemical change with melatonin (no change in BP) |

**Table S2:** NAESS exempt from reporting if **unrelated to IMP administration**

| **Hypoxic Ischemic encephalopathy (HIE):** Injury to the central nervous system that occurs when there is insufficient delivery of oxygen to all or part of the brain. | | | |
| --- | --- | --- | --- |
| Grade 3 | Severe | | Moderate clinical signs* of encephalopathy resulting from a perinatal asphyxia event; meeting criteria for therapeutic hypothermia.  Note: *see Sarnat stages for further guidance on mild, moderate and severe clinical signs of encephalopathy. |
| Grade 4 | Life  Threatening | | Severe clinical signs* of encephalopathy resulting from a perinatal asphyxia event with life-threatening consequences (e.g. respiratory depression, refractory seizures).  Note: *see Sarnat stages for further guidance on mild, moderate and severe clinical signs of encephalopathy. |
| **Neonatal Convulsion:** Sudden, involuntary, rapid rhythmic or stereotyped skeletal muscular contraction in a newborn | | | |
| Grade 3 | Severe | | Suspected seizures uncontrolled with 1 anti-seizure drug (recurrence within 3 days after treatment or requiring 2 or more anti-seizure drugs). |
| **Neonatal Epileptic Seizure**: An EEG confirmed paroxysmal surge of electrical activity in the brain that may result in physical or behavioural changes in a neonate | | | |
| Grade 3 | Severe | | EEG-proven seizures* uncontrolled with 1 anti-seizure drug (recurrence within 3 days after treatment or requiring 2 or more anti-seizure drugs).  Note: *Electrographic confirmation required: can be electro-clinical or electrographic only. |
| **Infant Sedation:** A state of a lowered level of consciousness. | | | |
| Grade 3 | Severe | | Severe sedation, major changes in feeding behaviour; requiring support other than oral feeding (e.g. tube feeding). |
| **Infant Irritability:** Crying easily, difficult to console. | | | |
| Grade 3 | Severe | | Severe irritability, major changes in feeding behaviour, requiring support other than oral feeding; requiring long term medical treatment (e.g. sedatives). |
| **Neonatal Sinus Bradycardia:** An abnormally low heart rate for age. | | | |
| Grade 3 | Severe | Persistent bradycardia; resulting in non-life-threatening hemodynamic compromise; requiring major care changes (e.g. new medication or intervention). | |
| **Neonatal Oedema:** Accumulation of an excessive amount of fluid in cells or intercellular tissues. | | | |
| Grade 3 | Severe | | Severe oedema; limiting age-appropriate behaviour; requiring major care changes (e.g. diuretics). |
| **Infantile Apnoea:** Cessation of air flow. | | | |
| Grade 3 | Severe | | Apnoea requiring stimulation and sustained FiO2 increase; requiring non-invasive ventilation; reoccurrences requiring start of or relevant increase in dose of respiratory stimulants or other major care changes. |
| **Neonatal Respiratory Insufficiency:** Significant impairment of gas exchange resulting in compensatory breathing efforts and eventually hypoxia and/or hypercarbia. *  Note: *Consider this adverse event for any patient whose respiratory condition deteriorates from baseline. If any specific diagnosis is identified: use diagnosis specific scale (e.g. RDS, pulmonary haemorrhage) | | | |
| Grade 3 | Severe | | Clinical evidence of increased respiratory distress with relevant deterioration in gas exchange (increase in pCO2 or decrease in oxygenation); requiring major care change (e.g. start invasive support). |
| **Feeding Intolerance:** Inability to achieve a full feeding volume. | | | |
| Grade 3 | Severe | | Severe feeding intolerance: requiring major changes in feeding support indicated (e.g. change to TPN or gavage). |
| **Neonatal Coagulation Disorder:** A condition of abnormal blood clotting or bleeding. | | | |
| Grade 3 | Severe | | Biochemical or clinical coagulation abnormalities; requiring intervention. |

**Table S3:** Detailed Schedule of Events

| **Schedule of Events** | **Screening** | **T0** | **T0+**  **2h** | **T0+**  **24h** *(±1h)* | **T0+**  **26h** | **T0+**  **36h**  *(±1h)* | **T0+**  **48h**  *(±1h)* | **T0+**  **60h**  *(±1h)* | **T0+**  **72h**  *(±1h)* | **T0+**  **84h**  *(±1h)* | **T0+**  **96h**  *(±1h)* | **T0+ Day 7**  (±1 day) | **T0+  Day 10** | **Hospital Discharge** | **90 days**  (± 7 days) |
| --- | --- | --- | --- | --- | --- | --- | --- | --- | --- | --- | --- | --- | --- | --- | --- |
| Informed Consent | X |  |  |  |  |  |  |  |  |  |  |  |  |  |  |
| Eligibility | X |  |  |  |  |  |  |  |  |  |  |  |  |  |  |
| Clinical Stability | X | X |  | X |  | X | X | X | X | X |  |  |  |  |  |
| Baby Demographics | X |  |  |  |  |  |  |  |  |  |  |  |  |  |  |
| Maternal Characteristics | X |  |  |  |  |  |  |  |  |  |  |  |  |  |  |
| Pregnancy and Delivery History | X |  |  |  |  |  |  |  |  |  |  |  |  |  |  |
| Placental Histology | X |  |  |  |  |  |  |  |  |  |  |  |  |  |  |
| Physical Examination | X |  |  |  |  |  |  |  |  |  |  |  |  |  | X |
| Therapeutic Hypothermia | X | X |  | X |  | X | X | X | X | X |  |  |  |  |  |
| Vital Signs | X | Please refer to table 3 | | | | | | | | | X |  |  |  | X |
| Cranial Ultrasound with Resistance Index | X |  |  | X |  |  | X |  | X |  | X |  |  |  |  |
| aEEG/EEG | X | X |  | X |  | X | X | X | X | X | X |  |  |  |  |
| NIRS | X | X |  | X |  | X | X | X | X | X | X |  |  |  |  |
| Modified Sarnat Neurological Examination | X |  |  | X |  |  | X |  | X |  | X | X |  | X |  |
| Concomitant Medications | X | X |  | X |  | X | X | X | X | X | X | X | X | X | X |
| Review of AE and SAEs | X | X |  | X |  | X | X | X | X | X | X | X | X | X | X |
| DLE Safety Monitoring |  | Continuous DLE monitoring from T0 to 100-hours after the final IMP administration | | | | | | | | | | |  |  |  |
| Cord/Blood Gas pH/Base Deficit | X |  |  |  |  |  |  |  |  |  |  |  |  |  |  |
| Biochemistry *(Renal, Liver Profile, CRP, Troponin, CK, LDH*) | X |  |  | X |  |  | X |  | X |  | X |  |  |  | X |
| Blood Culture | X |  |  |  |  |  |  |  |  |  |  |  |  |  |  |
| Blood Gas | X |  |  | X |  |  | X |  | X |  | X |  |  |  |  |
| Full Blood Count | X |  |  | X |  |  | X |  | X |  | X |  |  |  | X |
| Clotting including Fibrinogen | X |  |  |  |  |  |  |  |  |  |  |  |  |  |  |
| IMP Administration |  | X |  | X |  | X | X | X | X |  |  |  |  |  |  |
| PK Blood Sample |  | X | X | X | X |  | X |  |  |  | X |  |  |  |  |
| MRI/MRS Neuroimaging |  |  |  |  |  |  |  |  |  |  | X | | |  |  |
| Hammersmith Neonatal Neurological Examination (HNNE) |  |  |  |  |  |  |  |  |  |  |  |  |  | X |  |
| Hearing Screen |  |  |  |  |  |  |  |  |  |  |  |  |  | X |  |
| Discharge Characteristics |  |  |  |  |  |  |  |  |  |  |  |  |  | X |  |
| General Movement Assessment |  |  |  |  |  |  |  |  |  |  |  |  |  | X | X |
| Hammersmith Infant Neurological Examination (HINE) |  |  |  |  |  |  |  |  |  |  |  |  |  |  | X |
| Ages and Stages Questionnaire (ASQ-3) |  |  |  |  |  |  |  |  |  |  |  |  |  |  | X |
